# Controllable Room-Temperature Synthesis of Highly Stable CsPbBr_3_ Perovskite Quantum Dots via Synergistic Optimization of Br/Pb and OA/OAm Ratios

**DOI:** 10.3390/molecules31061006

**Published:** 2026-03-17

**Authors:** Yiting He, Xiayu Zhu, Ajun Li, Shuyuan Lin, Bo Li, Songbin Liu, Xinyu Ye

**Affiliations:** 1 College of Rare Earths, Jiangxi University of Science and Technology, Ganzhou 341000, China; 2 Key Laboratory of Testing and Tracing of Rare Earth Products for State Market Regulation, Jiangxi University of Science and Technology, Ganzhou 341000, China; 3 Key Laboratory of Low Dimensional Quantum Materials and Sensor Devices of Jiangxi Education Institutes, Jiangxi University of Science and Technology, Ganzhou 341000, China; 4 Key Laboratory of Testing and Tracing of Rare Earth Products, State Administration for Market Regulation, Ganzhou 341000, China

**Keywords:** CsPbBr_3_, perovskite quantum dots, Br/Pb precursor ratio, OA/OAm ratio

## Abstract

CsPbBr_3_ perovskite quantum dots (QDs) have attracted significant attention for optoelectronic applications owing to their outstanding optical properties, yet achieving controlled synthesis with high stability under mild conditions remains a challenge. The room-temperature synthesis of CsPbBr_3_ perovskite quantum dots using a coprecipitation method is systematically investigated in this work, with an emphasis on how the structural and optical properties of the QDs are influenced by the Br/Pb ratio and OA/OAm ratio. The findings show that controlling the Br/Pb and OA/OAm ratios can effectively influence the size, crystalline phase, and surface passivation properties of CsPbBr_3_ quantum dots. The photoluminescence peak shifts blue and the bandgap widens when the Br/Pb ratio rises due to a decrease in quantum dot size. This is mainly explained by more effective surface covering by Br^−^ ions and increased quantum confinement effects. The resultant quantum dots demonstrate ideal optical performance at a Br/Pb ratio of 75 and an OA/OAm ratio of 1.5, with dense ligand coverage, superior defect passivation, and markedly improved stability under UV irradiation and in aqueous environments. Variations in the Br/Pb and OA/OAm ratios affect the binding configuration and coverage of ligands on the quantum dot surface, thereby influencing the relationship between non-radiative recombination and the quantum confinement effect. The LED fabricated with the as-synthesized high-performance quantum dots demonstrates a wide color gamut, covering 129.45% of the NTSC standard, indicating strong potential for display applications.

## 1. Introduction

In recent years, metal halide perovskites have attracted significant attention from both academia and industry due to their exceptional physical properties and outstanding photovoltaic performance. Among these materials, inorganic perovskite quantum dots (QDs) are particularly distinguished by their strong quantum confinement effects, high photoluminescence quantum yields (PLQYs), and defect-tolerant structures [1,2,3]. Unlike many conventional semiconductor QDs, inorganic perovskite QDs maintain high luminescence efficiency even in the presence of high defect densities. Stemming from quantum confinement, they exhibit intense and tunable photoluminescence across the full visible spectrum, narrow full-width at half maximum (FWHM), large multiphoton absorption cross-sections, and low particle inversion thresholds [4,5]. These attributes enable their widespread application in light-emitting diodes (LEDs), solar cells, photodetectors, nonlinear optical sources, electro-optic modulators, and fast X-ray scintillators for ionizing radiation detection [6,7,8,9,10].

Inorganic perovskites primarily crystallize in three polymorphs, namely cubic, tetragonal, and orthorhombic, each offering substantial potential for diverse technological applications [11]. Notably, the thermodynamically favored phase at room temperature is closely correlated with the size of the perovskite QDs, and their structural stability is primarily governed by the Goldschmidt tolerance factor (TF), which is calculated as $TF=\frac{\left(R_{A}+R_{X}\right)}{\sqrt{2(R_{Pb}+R_{X})}}$ [12], where *R_A_*, *R_Pb_* and *R_X_* represent the ionic radii of the A-site cation, lead, and halide, respectively. For all-inorganic perovskites, the excessively small ionic radius of cesium relative to the A-site leads to thermodynamic instability [13]. In an ideal close-packed configuration, the tolerance factor equals 1. The TF value for CsPbBr_3_ is approximately 0.9, indicating structural instability [14]. At the nanoscale, perovskite quantum dots exhibit a high specific surface area, resulting in numerous surface metal atoms (e.g., Pb^2+^) that tend to form dangling bonds [15]. Meanwhile, bromine ions on the quantum dot surface are prone to detach from the lattice, generating halide vacancies. During solution processing or device fabrication, surface ligands often desorb, exposing these vacancy defects and thereby promoting non-radiative recombination and quantum dot aggregation. Such defect states, including lead vacancies, halide vacancies, and uncoordinated lead ions, act as non-radiative recombination centers that effectively trap photogenerated charge carriers. This trapping reduces the photoluminescence quantum yield (PLQY) and induces fluorescence blinking, limiting the applicability of perovskite quantum dots in high-brightness displays and high-sensitivity imaging [16,17]. Furthermore, these defects are major contributors to the structural instability of quantum dots. Under environmental stressors such as light exposure, elevated temperatures, oxygen, and moisture, they facilitate phase transitions, degradation, or agglomeration, leading to deteriorated optical performance and a substantially shortened operational lifetime [18,19]. Common methods for passivating defects include ion doping and ligand modification. Yieon Park et al. [20] proposed using trivalent metal halide doping to passivate perovskite surface defects and improve device stability. Qi Jiang et al. [21] introduced a post-treatment of perovskite surfaces with long-chain organic ammonium salts, which interact strongly with uncoordinated Pb^2+^ ions or halide vacancies. This strategy effectively passivates defects at surfaces and grain boundaries, thereby suppressing non-radiative recombination.

The phase stability of inorganic perovskites is highly sensitive to environmental factors, and the synthesis method significantly influences the crystal phase and size of QDs, thereby governing their quantum confinement, carrier transport, and luminescent properties [4,22]. In 2015, Protesescu et al. [23]. pioneered the hot-injection method to synthesize cubic-phase CsPbX_3_ QDs, systematically examining the effects of precursor ratios and reaction temperature. Schmidt et al. [24] later introduced the ligand-assisted reprecipitation (LARP) method for MAPbX_3_ QDs, valued for its simplicity and mild conditions. Subsequently, Li et al. [25] achieved room-temperature synthesis of monoclinic CsPbBr_3_ QDs, a phase distinct from hot-injection products. Solid-phase synthesis, first reported by Jana et al. [26], offers a solvent-free mechanochemical route for grinding CsBr, PbBr_2_, and n-octylammonium bromide at room temperature, but the resulting CsPbBr_3_ QDs achieved only 13% PLQY, which is significantly lower than that achieved by solution-based methods. Chemical vapor deposition (CVD) provides another effective route, growing QDs in tube furnaces under argon flow using CsX and PbX_2_ precursors, and yielding QDs with excellent optical performance widely used in photodetectors. These diverse strategies collectively enable tunable control over the structural and optical properties of inorganic perovskite QDs, supporting their broad optoelectronic applications [27].

As mainstream methods for inorganic perovskite QDs, existing techniques each have notable limitations. The hot-injection method achieves high photoluminescence quantum yields exceeding 90% but requires harsh conditions, complex operation, and suffers from poor reproducibility and scalability [28,29]. Solid-phase ball milling is solvent-free, environmentally friendly, and easily scalable, yet it introduces impurities and lattice defects, resulting in low optical performance and added process costs from post-annealing [30]. Chemical vapor deposition enables direct fabrication of uniform, high-purity films compatible with device integration, but demands high equipment investment, stringent conditions, and offers limited size control and low efficiency [31]. In contrast, the room-temperature co-precipitation method combines mild conditions, simple operation, low cost, good reproducibility, and easy halogen regulation [32,33]. Although its initial products show slightly lower crystallinity and quantum yield with broader size distribution compared to hot injection, these issues are readily addressed by ligand optimization or brief annealing, achieving near-equivalent performance [34]. Overall, this approach offers the most practical and controllable pathway for both scientific research and industrial applications.

In conventional room-temperature synthesis, the ratio of Oleic acid (OA) to oleylamine (OAm) serves as a critical parameter for regulating particle size, morphology, and optical properties of QDs. OA primarily functions as a solvent and stabilizer for the lead source by coordinating with Pb^2+^ ions to form lead oleate, thereby preventing premature precipitation of lead ions in polar environments and controlling the monomer concentration during the initial reaction stage [35]. In contrast, OAm provides steric hindrance through its long alkyl chains, effectively inhibiting excessive aggregation and growth of nanocrystals. Additionally, its amino groups (-NH_2_) form strong interactions with Br^−^ ions on the quantum dot surface, enabling timely passivation of surface bromine vacancies that readily form during room-temperature synthesis [36]. Nonetheless, systematic investigation is still needed as to how precursor dosages and ligand ratios affect the structure and properties of the resulting QDs, due to the absence of systematic investigation into the optimization of quantum dot performance.

This study employed a room-temperature precipitation method to synthesize perovskite quantum dots, using cesium acetate as the cesium source, lead acetate trihydrate as the lead source, and hydrobromic acid as a supplementary bromine source. The effects of precursor dosage and ligand ratio on quantum dot size, optical properties, and stability were systematically investigated, and their stability under different environmental conditions was quantitatively analyzed. The findings offer critical insights and provide a theoretical basis for the synthesis of high-performance perovskite quantum dots. Furthermore, the incorporation of the as-prepared quantum dots into LED encapsulation demonstrates their potential for use in optoelectronic devices.

## 2. Results and Discussion

### 2.1. The Effect of the Cs-Pb Ratio on Quantum Dot Properties

**Figure 1:**
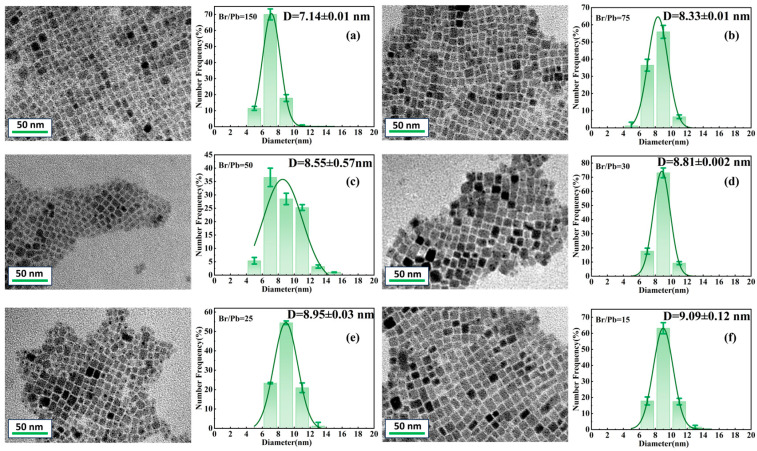
TEM and Particle Size Analysis of CsPbBr_3_ QDs with Different Br/Pb Ratios: (**a**) Br/Pb = 150; (**b**) Br/Pb = 75; (**c**) Br/Pb = 50; (**d**) Br/Pb = 30; (**e**) Br/Pb = 25; (**f**) Br/Pb = 15.

Figure 1a–f present transmission electron microscopy (TEM) images of CsPbBr_3_ QDs synthesized with varying Br concentrations, along with corresponding particle size distribution analyses. The TEM images reveal that the QDs predominantly exhibit a cubic or pseudo-cubic morphology, indicating consistency with the typical crystal structure of perovskites. Grain size distribution curves were obtained by analyzing 150 individual grains from different regions of quantum dots synthesized with varying Br/Pb ratios, using particle size analysis software. During room-temperature synthesis, as the bromine-to-lead ratio decreases, the size of the quantum dots gradually increases. This trend is attributed to the enhanced surface coverage of Br^−^ ions at higher Br/Pb ratios, which reduces the surface energy. To maintain energetic equilibrium, the QDs increase their specific surface area by reducing in size, thereby stabilizing the surface energy [37]. Meanwhile, Br^−^ ions also function as halide species that act as surface ligands or modulate the nucleation rate during synthesis. A high Br concentration may promote rapid nucleation, leading to the formation of a larger number of smaller QDs. Additionally, Br^−^ can coordinate with Pb^2+^ to form complexes, thereby inhibiting crystal growth and resulting in a reduced final particle size. In contrast, under low Br concentration conditions, QDs are more prone to aggregation or continued growth, leading to increased particle sizes and, in some cases, a phase transition toward an orthorhombic structure [38,39]. While a high Br/Pb ratio favors the formation of small-sized QDs with excellent monodispersity, it may compromise structural stability due to the increased likelihood of Br^−^ detachment from the surface. Conversely, a low Br/Pb ratio yields larger QDs but with a broader size distribution, the monodispersity of which can be improved by optimizing reaction parameters such as temperature, precursor concentration, or ligand species.

**Figure 2:**
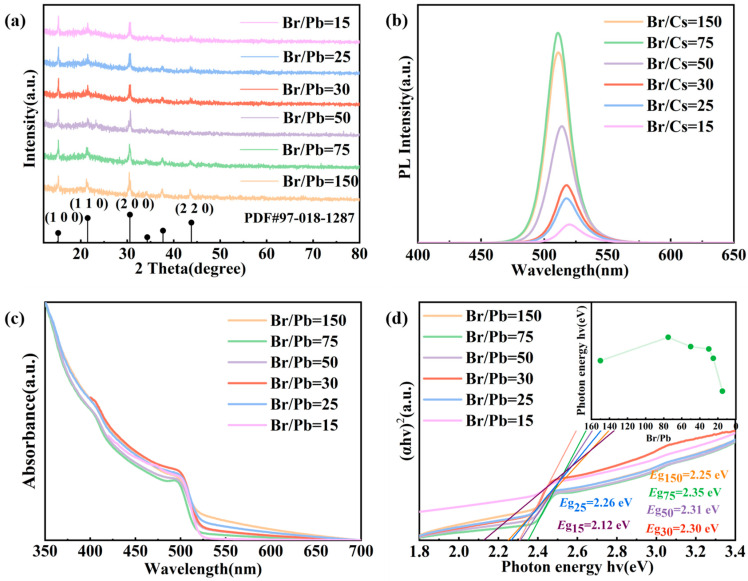
(**a**) XRD patterns; (**b**) PL spectra; (**c**) UV-vis absorption spectra; and (**d**) corresponding bandgap energies of CsPbBr_3_ QDs synthesized with different Br/Pb molar ratios.

To further investigate the effect of varying Br/Pb ratios on the structural characteristics of the QDs, X-ray diffraction (XRD) analysis was performed, with the results shown in Figure 2a. The XRD patterns of all samples are consistent with the characteristic peak positions of the standard card PDF#97-018-1287, confirming that the as-prepared QDs are phase-pure. As the Br/Pb ratio increases, the full-width at half maximum (FWHM) of the diffraction peaks gradually broadens, which is attributed to the reduction in quantum dot size—a trend consistent with the particle size evolution observed by TEM. Moreover, in the vicinity of the (200) diffraction peak, a gradual splitting into a doublet is observed with decreasing Br/Pb ratio. This phenomenon is ascribed to the increased quantum dot size at lower Br/Pb ratios, which induces a partial phase transition from the cubic to the orthorhombic structure.

The photoluminescence (PL) spectra, absorption spectra, and corresponding bandgap variations in QDs synthesized with different Br/Pb ratios are presented in Figure 2b–d. Comprehensive analysis reveals that the optical properties of the QDs exhibit systematic changes with varying Br/Pb ratios. As the Br/Pb ratio increases, the PL intensity initially rises, reaching a maximum at Br/Pb = 75, and subsequently declines. This trend is accompanied by a blue shift in the PL peak and a progressive widening of the bandgap. These phenomena are attributed to the gradual reduction in quantum dot size with increasing Br/Pb ratio: smaller particle dimensions lead to a broader bandgap due to enhanced quantum confinement. When the particle size approaches the exciton Bohr radius, surface ligands effectively passivate defects, suppress non-radiative recombination, and thereby promote high PL efficiency [40]. Conversely, excessively large particle sizes may introduce structural defects or weaken quantum confinement, ultimately reducing luminescence efficiency [41]. Notably, the exciton Bohr radius of CsPbBr_3_ QDs is approximately 7 nm [42]. At a low Br/Pb ratio of 15, the average particle size is relatively large (approximately 9.09 nm), exceeding this radius and resulting in weakened quantum confinement. Consequently, the bandgap approaches the bulk material value (approximately 2.25 eV), and non-radiative recombination channels become more prevalent. As the Br/Pb ratio increases from 15 to 75, the quantum dot size decreases progressively and approaches the Bohr radius. Within this regime, surface ligands passivate defects more effectively, suppressing non-radiative centers and significantly enhancing photoluminescence. At the optimal ratio of Br/Pb = 75, the particle size reaches approximately 8.33 nm, slightly above the Bohr radius yet still within a strongly coupled regime, where a favorable balance between quantum confinement and surface passivation is achieved. However, when the Br/Pb ratio exceeds 75, further size reduction leads to a sharp increase in the proportion of surface atoms and a rise in undercoordinated dangling bonds, which introduces additional surface trap states and consequently diminishes PL intensity. This interpretation is further supported by the observed trends in bandgap variation.

**Figure 3:**
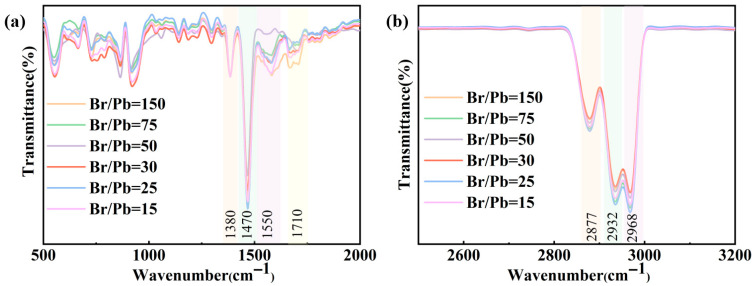
FTIR spectra of CsPbBr_3_ QDs synthesized with different Br/Pb ratios: (**a**) 500–2000 cm^−1^ region and (**b**) 2500–3200 cm^−1^ region.

To investigate the surface chemical mechanisms underlying the variation in photoluminescence intensity with different Br/Pb ratios, Fourier transform infrared (FTIR) spectroscopy was performed on the samples. The symmetric deformation vibration of the terminal -CH_3_ groups in the alkyl chains at approximately 1380 cm^−1^ is insensitive to the coordination environment and surface chemistry, and this peak is relatively free from interfering signals, making it easily identifiable [43,44]. Therefore, it was used as an internal reference for normalization, as shown in Figure 3a,b. All samples exhibited characteristic C-H stretching vibrations at approximately 2932 cm^−1^ and 2877 cm^−1^, confirming the presence of OA and OAm ligands [25]. The Infrared Oscillating Peak at approximately 1470 cm^−1^ and 1550 cm^−1^ corresponds to the symmetric and asymmetric stretching vibrations of carboxylate (COO^−^), respectively, while the peak at approximately 1710 cm^−1^ is attributed to uncoordinated carboxylic acid groups (-COOH) from free oleic acid [39]. At higher Br/Pb ratios, excess Br^−^ ions tend to occupy surface sites and may compete with carboxylate groups for adsorption [25], resulting in a relatively intense peak at 1710 cm^−1^. As the Br/Pb ratio decreases, oleic acid becomes fully deprotonated to form carboxylate (COO^−^), which coordinates with surface Pb^2+^ ions in either a bidentate or bridging mode. Meanwhile, the -NH_2_ groups of oleylamine interact with surface Br^−^ through hydrogen bonding or electrostatic attraction [45]. With a further reduction in the Br/Pb ratio, the increased particle size leads to a smaller specific surface area, thereby reducing the ligand demand. Under these conditions, some ligands may only be physically adsorbed onto the quantum dot surface, which also contributes to a pronounced peak at approximately 1710 cm^−1^.

**Figure 4:**
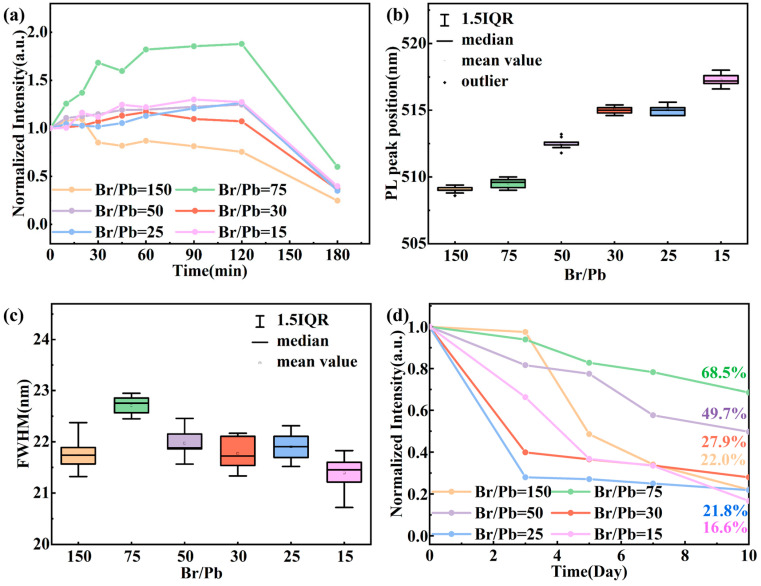
Time-dependent changes in QDs synthesized with different Br/Pb ratios: (**a**) PL intensity; (**b**) emission peak position; (**c**) full-width at half maximum (FWHM); and (**d**) PL intensity variation in quantum dot powders over 10 days of storage in water.

As shown in Figure 4a–c, the time-dependent photoluminescence (PL) intensity, peak position, and full-width at half maximum (FWHM) of QDs synthesized with different Br/Pb ratios were monitored under UV irradiation. All samples exhibited an initial increase followed by a decrease in PL intensity, a phenomenon attributed to the competing effects of photoinduced passivation and photodegradation. At the early stage of UV exposure, CsPbBr_3_ QDs possess numerous bromine vacancies on their surfaces, which act as trap states for charge carriers and serve as non-radiative recombination centers [46]. Under UV illumination, high-energy photogenerated electrons and holes facilitate reactions between adsorbed molecules and the quantum dot surface, either filling anion vacancies or capturing electrons from photoexcited QDs to form superoxide radicals (O_2_^−^), thereby neutralizing surface defects [47]. Additionally, the energy provided by UV light promotes the rearrangement of loosely bound or physically adsorbed OA and OAm ligands on the quantum dot surface. This reorganization enables more effective surface passivation, allowing excess ligands to bind to unsaturated Pb or Br sites. The reduction in surface defects combined with enhanced ligand passivation leads to a noticeable increase in PL intensity [48,49].

However, as UV irradiation continues, prolonged exposure to high-energy photons begins to disrupt the crystal lattice. Halide ions (Br^−^) undergo migration both within and between grains, resulting in the formation of bromide-rich and bromide-deficient regions over time. This ion migration progressively undermines the structural uniformity and optical homogeneity of the QDs, ultimately causing a decline in PL intensity accompanied by shifts in emission peak position and broadening of the FWHM. These changes reflect the gradual dominance of photodegradation mechanisms, leading to the deterioration of optical performance. The cumulative effect causes damage to exceed repair, leading to a decline in fluorescence [50].

Meanwhile, the evolution of photoluminescence under UV irradiation is significantly influenced by the Br/Pb ratio. At higher Br/Pb ratios, the QDs possess smaller sizes, larger specific surface areas, and higher surface reactivity, which facilitates the rapid adsorption and passivation of environmental molecules during the initial stage of illumination, resulting in a pronounced initial enhancement of PL intensity. However, the high surface energy also renders the ligands prone to detachment, leading to a subsequent rapid decline in PL. At the optimal ratio of Br/Pb = 75, a favorable balance between quantum confinement and surface passivation is achieved, yielding structurally stable QDs with enhanced overall stability. In contrast, at lower Br/Pb ratios, the QDs are larger with smaller specific surface areas and may contain more intrinsic lattice defects, which weakens the effectiveness of photoinduced passivation. Throughout the illumination process, neither the FWHM nor the emission peak position exhibited significant changes across all samples, indicating that while PL intensity fluctuates due to variations in surface states, the core crystal structure of the QDs remains stable without undergoing substantial phase transitions or size alterations. This further confirms that the photoinduced processes primarily modulate surface defects rather than the internal crystal structure.

To further investigate the stability of QDs synthesized with different Br/Pb ratios under environmental stress, water stability tests were conducted, with results shown in Figure 4d. As storage time in water increased from 0 to 10 days, the PL intensity of all samples exhibited a declining trend, attributable to water molecule erosion of the quantum dot surface, leading to ligand detachment and lattice hydrolysis. Notably, QDs synthesized with Br/Pb = 75 demonstrated the slowest decay curve, maintaining a relatively high PL intensity even after 10 days of water exposure. This observation further corroborates that the Br/Pb = 75 synthesis condition yields QDs with the most compact surface ligand coverage and minimal structural defects, providing the most effective barrier against water molecule infiltration.

### 2.2. Effect of OA to OAm Ratio on Quantum Dot Properties

**Figure 5:**
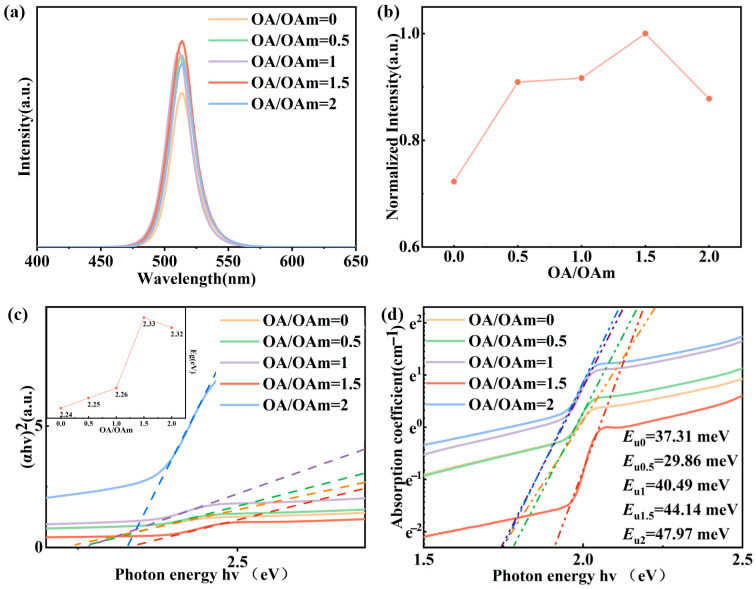
(**a**) PL spectra; (**b**) relative PL intensity; (**c**) bandgap energy; and (**d**) Urbach tail of CsPbBr_3_ QDs synthesized with different OA/OAm ratios.

To investigate the regulatory effect of the OA/OAm ratio on the properties of QDs, a series of samples with varying OA/OAm ratios were prepared while keeping the Br/Pb molar ratio constant at 75. The OA/OAm ratio ranged from 0 to 2, where OA/OAm = 0 corresponds to the synthesis of CsPbBr_3_ QDs without using OA. In a separate control experiment conducted without OAm, the resulting product exhibited significant precipitation and could not be dispersed in common solvents. This failure is attributed to the limited binding capacity and inherent instability of oleic acid when used alone; the ligand readily detaches from the QD surface, leading to severe aggregation. These observations confirm that the presence of OAm is essential for maintaining colloidal stability and dispersibility during the synthesis of CsPbBr_3_ QDs. Consequently, the gradient design was confined to OA/OAm ratios from 0 to 2, excluding the OAm-free condition. The basic optical properties of the resulting QDs were systematically characterized, as presented in Figure 5a–d. As the OA/OAm ratio increases, the photoluminescence intensity, bandgap, and Urbach energy initially increase and then decrease, indicating that the OA/OAm ratio enables further fine-tuning of quantum dot properties. When the OA/OAm ratio is too low, an excess of oleylamine may lead to surface over-etching or the formation of an excessively thick ligand layer, thereby introducing defects. Conversely, when the OA/OAm ratio is too high, an excess of oleic acid weakens the passivation capability of OAm toward halide vacancies, resulting in increased surface defects. An appropriate OA/OAm ratio not only effectively controls crystal growth to achieve uniform size distribution but also maximally passivates surface bromine vacancies, thereby suppressing non-radiative recombination and reducing the band tail states (Urbach tail). This brings the bandgap closer to the ideal value and achieves the highest luminescence efficiency. These results demonstrate that following optimization of the Br/Pb ratio, further regulation of the ligand ratio is a critical step toward realizing high-performance CsPbBr_3_ QDs.

**Figure 6:**
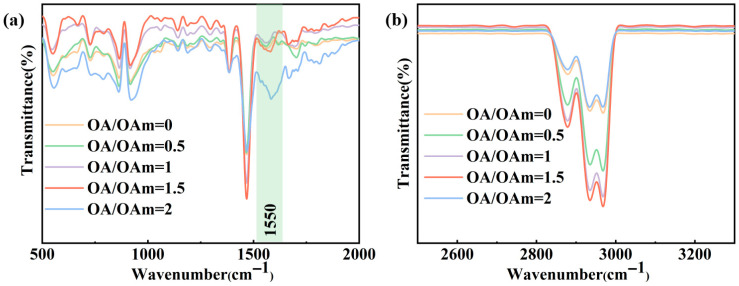
Fourier transform infrared (FTIR) spectra of CsPbBr_3_ QDs synthesized with different OA/OAm ratios: (**a**) 500–2000 cm^−1^ region and (**b**) 2500–3300 cm^−1^ region.

Figure 6 presents the FTIR spectra of QDs synthesized with different OA/OAm ratios, revealing variations in the surface ligand binding states under different OA/OAm conditions. As the OA/OAm ratio increases from 0 to 2, a slight shift is observed in the peak at approximately 1550 cm^−1^, indicating a change in the coordination mode between carboxylate groups (COO^−^) and surface Pb^2+^ ions. As shown in Figure 6b, the intensity of the characteristic C-H stretching vibrations of the alkyl chains (at approximately 2932 cm^−1^ and 2877 cm^−1^) first increases and then decreases with increasing OA/OAm ratio, reaching a maximum at OA/OAm = 1.5. Combined with the evolution of the coordinated COO^−^ peak at around 1550 cm^−1^, these results suggest that at OA/OAm = 1.5, OA and OAm form the most compact mixed ligand layer on the quantum dot surface, achieving the highest surface coverage. This densely packed ligand shell effectively shields the inorganic core from environmental degradation and maximally passivates surface defects. In contrast, either too low or too high OA/OAm ratio leads to reduced surface ligand coverage, as evidenced by the diminished C-H peak intensities, resulting in decreased quantum dot stability and increased non-radiative recombination. These trends are fully consistent with the observed variations in photoluminescence intensity and Urbach energy.

**Figure 7:**
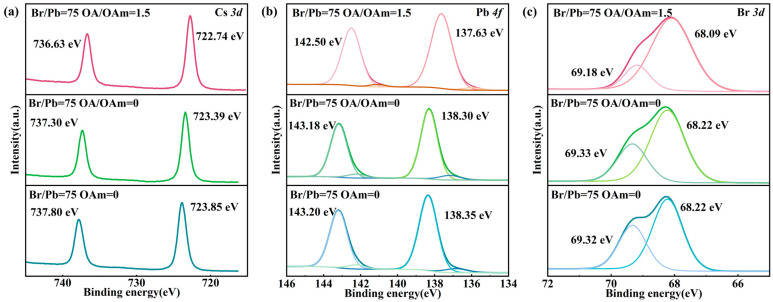
XPS spectra of quantum dots synthesized with a fixed Br/Pb ratio of 75 under different ligand conditions: OA/OAm ratio of 0 and 1.5, and in the absence of OAm: (**a**) Cs 3d; (**b**) Pb 4f; and (**c**) Br 3d.

XPS analysis was conducted on quantum dots with a Br/Pb ratio of 75 to investigate surface defects and ligand–QD interactions. Three types of samples were examined: liquid-phase QDs prepared with OA/OAm ratios of 1.5 and 0, and a powder sample obtained in the absence of OAm. The results are presented in Figure 7. Pb 4f high-resolution XPS spectra were examined. A shift in the Pb 4f peak toward lower binding energy was observed, indicating that unsaturated Pb^2+^ sites on the surface accepted electron pairs from nitrogen or oxygen atoms in OA and OAm, thereby passivating defect states [51]. In contrast, for QDs synthesized with OA/OAm = 0 and in the absence of OAm, a shoulder peak or asymmetric tail appeared on the low-binding-energy side, which is typically attributed to metallic Pb^0^, indicating severe surface defects [52]. For QDs synthesized with a Br/Pb ratio of 75 and an OA/OAm ratio of 1.5, the metallic Pb^0^ signal was significantly attenuated, confirming the effective passivation of uncoordinated Pb^2+^ sites by OA and OAm ligands. High-resolution Br 3d XPS spectra were analyzed. Compared to QDs synthesized with OA/OAm = 0 or without OAm, the optimal sample exhibited a distinct shift in the Br 3d peak toward lower binding energy. This shift indicates that Br^−^ ions reside in a higher electron density environment, confirming effective passivation of bromine vacancies [53]. For QDs synthesized without OAm, the Br 3d spectrum exhibited a more pronounced doublet feature, indicating that the absence of oleylamine leads to an unstable bromine chemical environment, where bromine vacancies or surface bromine species are not effectively passivated [45].

**Figure 8:**
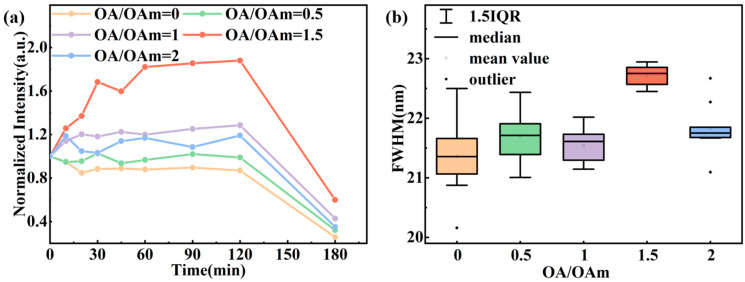
(**a**) Time-dependent PL intensity and (**b**) FWHM variation in CsPbBr_3_ QDs synthesized with different OA/OAm ratios under UV irradiation.

To further investigate the effect of OA/OAm ratio on the fine-tuning of quantum dot properties, their stability under UV irradiation was investigated, with results shown in Figure 8. Under continuous UV exposure, the photoluminescence intensity of all samples exhibited a synergistic evolution involving both photoinduced passivation and photodegradation. Throughout the illumination process, the FWHM remained stable at approximately 22.5 nm without significant variation, confirming that the core crystal structure of the QDs remained intact under light exposure and that the observed PL fluctuations primarily originated from changes in surface states. At the optimal OA/OAm ratio of 1.5, the surface ligands formed the most densely packed configuration, yielding the best photostability. Deviation from this optimal ratio resulted in reduced ligand coverage and accelerated photodegradation. These findings further underscore the critical importance of optimizing the OA/OAm ratio for achieving CsPbBr_3_ QDs with high stability.

### 2.3. Applications of the QDs in LED

**Figure 9:**
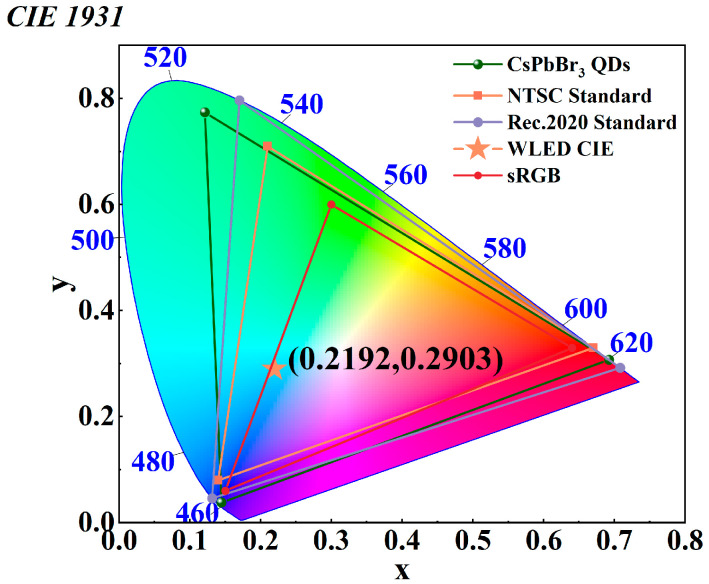
CIE color coordinates and color gamut diagram.

Leveraging QDs’ high PL efficiency and superior narrowband green emission, the as-prepared CsPbBr_3_ QDs were integrated with blue LED chips and red-emitting K_2_SiF_6_: Mn^4+^ phosphors to fabricate light-emitting diodes (LEDs). This configuration enabled an assessment of the practical applicability of the synthesized quantum dots in lighting and display technologies. Under a driving current of 20 mA, the resulting device exhibited CIE 1931 chromaticity coordinates of (0.2192, 0.2903) and possesses a high color rendering index (CRI = 79.03). As illustrated in the CIE chromaticity diagram (Figure 9), the LED demonstrated a broad color gamut. Based on the emission spectra of the red, green, and blue primaries, the color gamut coverage was calculated to be approximately 182.77% of the sRGB standard, 129.45% of the NTSC standard, and 96.66% of the ultra-high-definition Rec. 2020 standard. These findings collectively underscore the significant potential of the as-synthesized CsPbBr_3_ QDs for the fabrication of high-performance, wide-color-gamut display devices.

## 3. Experimental Section

### 3.1. Raw Materials

All chemicals were used as received: Cesium Ethanoate (CsAc, 99.99%, Aladdin (Shanghai, China)), Lead acetate trihydrate (Pb(OAc)_2_·3H_2_O, ≥99.99% metals basis, Aladdin), oleic acid (OA, technical grade 90%, Aladdin), Oleylamine (OAm, ≥98% (primary amine), Sigma-Aldrich (Shanghai, China)), Acetic acid (HAc, ≥99%, Aladdin), Hydrobromic acid (HBr, ≥99.99% metals basis, Aladdin), Toluene (TL, AR, Aladdin), and N-hexane (anhydrous, Aladdin).

### 3.2. Synthesis of CsPbBr_3_ QDs

The precursor solution was prepared by dissolving 0.2 mmol of cesium acetate (CsAc) and 2 mmol of lead acetate trihydrate (Pb(OAc)_2_·3H_2_O) in 5 mL of acetic acid under ambient conditions. The mixture was stirred until complete dissolution of the salts, yielding a clear and homogeneous solution. In a separate reaction vessel, the anti-solvent mixture was formulated by combining 20 mL of toluene, 1.5 mL of OA, 1.0 mL of OAm, and 6 mmol of hydrobromic acid (HBr). This mixture was magnetically stirred for 5 min to ensure uniform dispersion of all components. Under continuous magnetic stirring, a certain amount of the precursor solution was rapidly injected into the pre-stirred anti-solvent system. The reaction was allowed to proceed for 1 min, during which CsPbBr_3_ perovskite QDs were formed in situ via anti-solvent-induced rapid precipitation.

Purification: The QDs were washed using n-hexane as the washing solvent. Centrifugation was performed at 8000 r/min for 10 min to remove the supernatant, thereby eliminating excess ligands from the QDs. Subsequently, 5 mL of n-hexane was added, followed by centrifugation at 4000 r/min for 5 min to discard the precipitate, which removed large-sized crystalline particles from the QDs. After repeated washing and purification steps, the resulting quantum dot solution was stored in a brown sample vial under refrigerated conditions at 0–5 °C.

CsPbBr_3_ quantum dot powder was prepared using an anti-solvent method. Ethanol was employed as the anti-solvent and added to the quantum dot toluene solution at a volume ratio of 2:1 (ethanol to solution). The resulting precipitate was collected through repeated cycles of washing and centrifugation. After drying, the final CsPbBr_3_ quantum dot powder was obtained. The prepared powder was stored in a glass vial within a desiccator for subsequent use.

### 3.3. QDs Characterization

To elucidate the effects of varying Br/Pb and OA/OAm ratios on the structural and optical properties of CsPbBr_3_ quantum dots, a comprehensive characterization was performed. UV-Vis absorption spectra were recorded using a PerkinElmer Lambda 35 spectrophotometer (PerkinElmer, Waltham, MA, USA) at room temperature under ambient air conditions. The crystal structures were characterized by X-ray diffraction (XRD) on a Malvern Panalytical Empyrean diffractometer (Malvern Panalytical B.V, Almelo, The Netherlands) with Cu Kα radiation, using samples prepared by drop-casting the liquid dispersion onto substrates to form thin films at room temperature. Fourier-transform infrared (FTIR) spectra were measured using a Bruker ALPHA II spectrometer (Bruker Corporation, Billerica, MA, USA) at room temperature under ambient air conditions. Transmission electron microscopy (TEM) images were acquired using an FEI Tecnai G2 F20 microscope (Thermo Fisher Scientific, Bend, OR, USA). Photoluminescence (PL) spectra were recorded with an Edinburgh Instruments FLS980 fluorescence spectrophotometer (Edinburgh Instruments, Livingston, UK) at an excitation wavelength of 365 nm under ambient air conditions at room temperature. The photostability under UV light was evaluated by continuously illuminating the samples with 365 nm UV light in a sealed container at room temperature and monitoring their emission using the Edinburgh Instruments FLS980 fluorescence spectrophotometer. The water stability was assessed by immersing the samples in water within a cuvette at room temperature for a specified duration and subsequently measuring their emission using the same Edinburgh Instruments FLS980 fluorescence spectrophotometer. Surface elemental composition of the hybrid material was examined by X-ray photoelectron spectroscopy (XPS) on a Thermo Scientific K-Alpha spectrometer (Thermo Fisher Scientific, Waltham, MA, USA) with Al Kα radiation. Liquid samples were drop-cast onto substrates at room temperature to form thin films prior to analysis.

## 4. Conclusions

This study systematically investigates the structure–property relationships of CsPbBr_3_ perovskite QDs synthesized via a room-temperature coprecipitation method by finely tuning the Br/Pb molar ratio and the OA/OAm ligand ratio. The results demonstrate that the Br/Pb ratio governs the size distribution, phase evolution, and optical bandgap of QDs by influencing particle size, surface Br^−^ coverage, and nucleation kinetics. In turn, the OA/OAm ratio further optimizes surface passivation and suppresses non-radiative recombination by modulating the packing density and coordination mode of surface ligands. The synergistic regulatory mechanism between the Br/Pb and OA/OAm ratios enables precise control over both quantum dot size and surface defects during room-temperature synthesis, establishing a reproducible parameter system for fabricating perovskite QDs with high brightness and excellent stability. This optimized strategy not only advances the practical application of QDs in optoelectronic devices and energy-related fields but also provides valuable insights into ligand-to-ion competitive adsorption, offering a useful reference for the surface engineering and controlled synthesis of other functional nanomaterials.

## Data Availability

The original contributions presented in this study are included in the article. Further inquiries can be directed to the corresponding authors.
